# Multimodal Art Pose Recognition and Interaction With Human Intelligence Enhancement

**DOI:** 10.3389/fpsyg.2021.769509

**Published:** 2021-11-08

**Authors:** Chengming Ma, Qian Liu, Yaqi Dang

**Affiliations:** College of Communication, Northwest Normal University, Lanzhou, China

**Keywords:** intelligent augmentation, multimodality, human ART, pose recognition, interaction

## Abstract

This paper provides an in-depth study and analysis of human artistic poses through intelligently enhanced multimodal artistic pose recognition. A complementary network model architecture of multimodal information based on motion energy proposed. The network exploits both the rich information of appearance features provided by RGB data and the depth information provided by depth data as well as the characteristics of robustness to luminance and observation angle. The multimodal fusion is accomplished by the complementary information characteristics of the two modalities. Moreover, to better model the long-range temporal structure while considering action classes with sub-action sharing phenomena, an energy-guided video segmentation method is employed. And in the feature fusion stage, a cross-modal cross-fusion approach is proposed, which enables the convolutional network to share local features of two modalities not only in the shallow layer but also to obtain the fusion of global features in the deep convolutional layer by connecting the feature maps of multiple convolutional layers. Firstly, the Kinect camera is used to acquire the color image data of the human body, the depth image data, and the 3D coordinate data of the skeletal points using the Open pose open-source framework. Then, the action automatically extracted from keyframes based on the distance between the hand and the head, and the relative distance features are extracted from the keyframes to describe the action, the local occupancy pattern features and HSV color space features are extracted to describe the object, and finally, the feature fusion is performed and the complex action recognition task is completed. To solve the consistency problem of virtual-reality fusion, the mapping relationship between hand joint point coordinates and the virtual scene is determined in the augmented reality scene, and the coordinate consistency model of natural hand and virtual model is established; finally, the real-time interaction between hand gesture and virtual model is realized, and the average correct rate of its hand gesture reaches 99.04%, which improves the robustness and real-time interaction of hand gesture recognition.

## Introduction

With the development and application of artificial intelligence, machine vision, human-computer interaction, and other technologies, computers are rapidly integrated into our lives and gradually change our lifestyles, in which human-computer interaction (HCI) technology, which allows close contact between humans and machines, continues to emerge as innovative and novel, which serves as a special language for communication between humans and computers, such as body movements, facial expressions, etc., to express specific interaction intentions and achieve the exchange of information between humans and computers (Abdullah et al., 2021). This kind of interaction not only restricts people’s actions, but also affects the interaction between people and computers. Furthermore, it gradually derives to the operation of autonomous actions by people, such as gestures, voice, body posture, facial expressions and so on. Initially, the traditional human-computer interaction is operated by external forces such as the mouse and keyboard, which not only constrains human behavior and action but also affects the interaction between humans and computers. Further, it is gradually derived to the operation of behavioral actions issued by humans autonomously, such as gestures, voice, body gestures, facial expressions, etc. This way makes the human-computer interaction interface change from traditional to intelligent, from graphical user interface and network user interface to multimodal and multimedia intelligent interaction interface, from unilateral human-computer interaction to the stage of cognitive understanding of human, from satisfying basic functions to attaching importance to user experience, making the intelligent human-computer interaction interface more natural and diverse (Ludl et al., 2020). Ignoring the students’ hands-on operation ability during the experiment, most of the virtual experiments are inseparable from the teacher’s guidance during the operation, resulting in the students’ lack of independent research ability. The virtual teaching method gradually adds augmented reality (AR) technology based on simulation technology, which realizes the transformation from two-dimensional virtual space to three-dimensional real space, which is conducive to improving students’ interest and enriching the experience (Islam and Iqbal, 2021). The virtual experiment classroom, taking the chemistry experiment classroom as an example, not only has the problems of difficult observation of students’ experimental results, tedious experimental steps, and difficult experimental operations, but also ignores the students’ hands-on ability in the experimental process, and most of the virtual experiments cannot be operated without the teacher’s guidance, leading to the lack of students’ ability to conduct independent research (Rastgoo et al., 2021). The main task of traditional human action recognition is to design a complete and effective algorithm so that the computer can determine the category of human action from a series of pictures or a video. The realization process mainly includes two parts: feature extraction and classifier classification. These problems cannot be solved by unimodal interaction in AR alone, and making the virtual-real interaction in virtual experiments more efficient and enhancing students’ feelings of the authenticity of experiments is the main goal at present. Therefore, for domestic secondary school students’ virtual chemistry experiments, there is an urgent need for a new experimental system with intelligent interaction, which not only has the characteristics of the normal operation of the experimental process, but also needs to meet the characteristics of experimental ease of operation, experimental ease of observation, and low cost, which is an intelligent experimental system (Jain et al., 2021).

Action recognition also plays a big role in sports, art performance, etc. In sports, analyzing the basic posture movements of athletes and comparing them with the standard movements, can help athletes to check the deficiencies and fill in the gaps to a great extent, and assist in training; while in art performance, especially in dance performance, the standard movements largely determine the effect of training and the success of the performance, human motion recognition can help performers to complete training and performance better through motion analysis. The main task of traditional human action recognition is to design a complete and effective algorithm to make the computer determine the human action category from a series of pictures or a video (Jegham et al., 2020). The implementation process mainly consists of two major parts: feature extraction and classifier classification. Although the traditional manually designed feature representation can accomplish the given recognition task better, due to its excessive reliance on the database itself, the designed features can only satisfy the recognition task of a single database, and cannot be effectively applied to multiple databases, lacking generalization ability (Xue et al., 2020). At the same time, in the context of information explosion and big data caused by the improvement of hardware performance and computing power, the traditional manual design feature method is even more unable to meet the task requirements because of its inability to handle massive data and then faces elimination.

To solve the problem of human action recognition, this paper focuses on three aspects of multimodal human action recognition, object recognition, and human-object interaction action recognition. Through theoretical demonstration and experimental results comparing multi-modal data and single-modal data, the validity and feasibility of the multi-modal action recognition algorithm are verified. The focus is on exploring the effect of multimodal fusion on the improvement of complex action recognition accuracy, the effect of constructing a subset of object shape features on the improvement of object recognition accuracy, and the effect of fusing different subsets of features from different modalities on the improvement of human-object interaction action recognition. In addition, the application of multimodal data in classical action recognition algorithms is reproduced. Firstly, a theoretical introduction is made for two classical action recognition algorithms. Later action recognition algorithms have been improved in terms of data processing and network structure, but the basic model theoretical basis remains unchanged. Secondly, the effectiveness and feasibility of multimodal action recognition algorithms are verified through theoretical arguments and experimental results comparing multimodal data and unimodal data. Finally, through visualization experiments, the defects of the traditional action recognition algorithm are found, and the foundation for further improvement of the algorithm is laid. After the initial recognition of the action by the action recognition network, the network fusion strategy module is also designed to give different fusion methods for various situations that may occur during the fusion of the two networks, avoiding the misjudgment of the target object or other redundant information due to the target detection network, which may affect the overall accuracy of the action recognition algorithm.

## Current Status of Research

Multimodal recognition includes recognition of modalities such as gesture, speech, somatic, and sensing devices, where sensing devices are more accurate in detecting human behavior and are relatively easy to interact with, and are generally used in combination with other modalities, including sensors that detect various user behaviors, Kinect, data gloves, stereo helmets, etc. (Rasipuram and Jayagopi, 2020). Gesture recognition and speech recognition are common areas of research and have become important components of perceptual user interfaces in accomplishing intelligent human-computer interaction functions that are highly structured, which makes gestures and speech an essential interaction modality in the field of human-computer interaction (Pandeya and Lee, 2021). In human-computer interaction, it is found that gesture recognition is not only affected by different contexts, multiple interpretations, and spatial and temporal changes, but also has unsolved problems until now due to the complex non-rigid nature of the human hand, while speech recognition is more susceptible to the influence of surrounding environmental factors and human factors, and has great challenges (Halfon et al., 2021). However, it is found that gesture recognition, speech recognition, and sensor sensing can complement each other, and the use of multi-modal interaction can better reduce the user’s operational burden and improve the efficiency of interaction.

Therefore, if a real breakthrough in intelligence is to be achieved and the problem of the excessive operational load of unimodal interaction is solved, there is an urgent need to apply the combination of gestures, speech, and sensors to complement each other to achieve a human-computer interaction environment with more natural and intelligent features. Multimodal interaction is a very dynamic and extensive research area, and many researchers have proposed multimodal information fusion methods in recent years, mostly through multimodal integration strategies or fusion models (Kunda, 2020). Overall, multimodal fusion methods are mainly classified by level into fusion at the data layer, model (feature) layer, and decision layer (Yoon et al., 2020). Fusion at the data layer is the fusion of the entire multimodal information data into a single feature vector, which is characterized by centralized analysis and fusion of unpreprocessed data. The fusion of the model layer is the feature extraction of multimodal information and then comprehensive analysis and processing of the extracted feature information, which is characterized by the compression of sizable information, real-time processing of feature information, and the flexibility to choose the location of fusion (Poibrenski et al., 2021). Compared with video and image action recognition, skeletal point data is more robust to lighting conditions, viewpoint transformations, and changes in human form. Moreover, in complex pure action recognition (action recognition without the influence of other objects), skeleton point data has an advantage that video data cannot match (Morales et al., 2021). Regarding the action recognition of skeletal points, the traditional method of manually designed features usually represents the motion of several human skeletal nodes as the action of the whole human body. Based on the 3D coordinate position information of these skeletal nodes, the motion information is characterized and extracted from the skeletal joints using algorithms such as the K nearest neighbor classification algorithm (KNN) and dynamic time regularization (DTW).

It is eventually fed into a designed implicit Markov model (HMM) or a support vector machine (SVM) for classification. However, due to the limitations and inefficiencies of traditional machine learning algorithms and the unreliability of early data sources. Skeletal point-based action recognition algorithms have not received much attention from researchers for a long time. Complex scenes include a range of uncontrollable scenes such as the background in which the person is in the video, the environment of the camera, etc. Complex scenes can lead to reduced recognition effectiveness and decreased recognition accuracy of existing action recognition algorithms. Common complex scenes include the intensity of lighting, changes in shooting perspective, object occlusion, and camera shake itself. It is this variety of complex factors that cause the action recognition technology to not achieve the theoretical results in the practical application process. Recognition algorithms that are robust to such complex environments are in urgent need of development. More effective recognition of similar actions with object influence is performed by incorporating a target detection network and a target association module. After the initial recognition of the action by the action recognition network, a network fusion strategy module is designed to give different fusion methods for various situations that may occur during the fusion of the two networks to avoid misjudgment of the target object or other redundant information due to the target detection network, which may affect the overall accuracy of the action recognition algorithm.

## Intelligent Augmented Multimodal Human Art Pose Recognition and Interaction Analysis

### Intelligent Augmented Multimodal Human Art Pose Recognition Algorithm Design

Video-based action recognition algorithms have also entered a rapid development path. The data type of action recognition has changed from the original single RGB image data to the current multi-modal data (Ou et al., 2020), including but not limited to RGB video data, depth video data, and infrared video data. Including but not limited to RGB video data, depth video data and infrared video data, this requires that the existing motion recognition algorithms not only be compatible with different basic network model structures, to find the best network model to achieve the expected recognition accuracy, but also It must also be able to process data of different modalities, and use the complementarity of the feature information of multi-modal data to complete the recognition task. This requires existing action recognition algorithms to be compatible not only with different basic network model structures to facilitate finding the best network model to achieve the expected recognition accuracy, but also to be able to handle data from different modalities and utilize the complementary nature of the feature information of multimodal data to accomplish the recognition task.


(1) $$\left\{ \begin{array}{c} V_{\left(i,f\right)}=\frac{J_{i}^{f}+J_{i}^{f-1}}{T}\\ V_{i}=\left(V_{\left(i,i\right)},\ldots ,V_{\left(i,f\right)}\right) \end{array}\right.$$

(2) $$L_{\left(e\,m\,g,k\right)}=\sum_{f\,\left(e\,m\,g,k\right)\in A_{\left(e\,m\,g,k\right)}}\left(f_{\left(e\,m\,g,n\right)}+C_{\left(e\,m\,g,k\right)}\right)$$

In this section, two classical action recognition algorithms and their principles, from which most of the existing algorithms derive their theoretical basis, are described in detail, while both algorithms can handle multimodal data well and possess the effect of complementing multimodal information. Finally, the shortcomings in the classical algorithms are also found and improvements are proposed through experimental replication. In addition, the theory of the algorithm proposed in this paper is also based on the classical algorithm and is optimized and improved on this basis to achieve better results. The principle of an action recognition algorithm based on the dual-stream network is fundamentally different from the C3D algorithm, which extracts Spatio-temporal features directly from video data by integrating spatial features and temporal features. The latter directly extracts spatio-temporal features from video data by integrating spatial and temporal features. The former designed two isomorphic networks to extract the appearance and motion characteristics of the data, respectively, and the clues of the motion characteristics are provided by the timing information of the video.


(3) $$\left\{ \begin{array}{c} f\,\left(x\right)=s\,i\,g\,n\,\left(\sum_{i=1}^{N}a_{i}\,y_{i}\,k\,\left(x_{i},x_{j}\right)-b\right)\\ s\,i\,g\,n\,\left(x\right)=\left\{ \begin{array}{c} -x>0\\ x=0\\ x\leq 0 \end{array}\right. \end{array}\right.$$

(4) $$\min\,\sum_{i=1}^{N}a_{i}+\frac{1}{2}\,\sum_{i=1}^{N}\sum_{j}^{N}a_{i}\,y_{j}\,a_{j}\,y_{i}\,k\,\left(x_{i}^{2},x_{j}^{2}\right)$$

**FIGURE 1:**
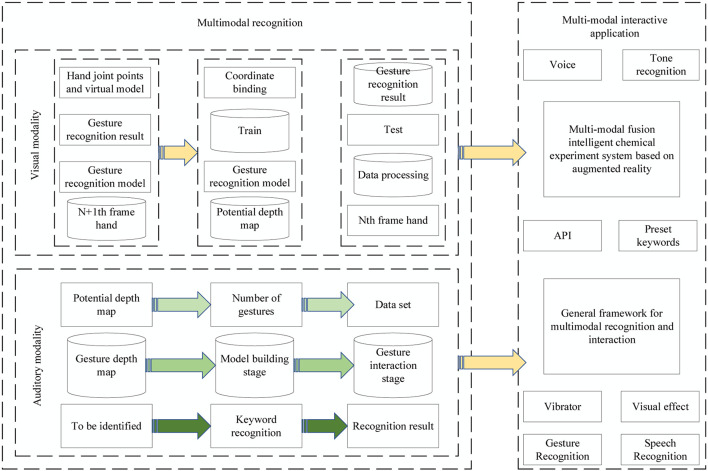
General framework for multimodal recognition and interaction.

In the spatial stream, the spatial network receives the single RGB image after frame splitting and outputs the judged action category by extracting the spatial information features of the image. Spatial information contains a variety of information such as complex background information, texture information, and shape information of the video, and doing information extraction for the pictures helps to recognize actions with the help of appearance information features of the human body and video background features. The recognition of visual modal is mainly divided into the establishment of gesture recognition model and gesture interaction stage. The recognition of voice modal is mainly keyword recognition. In addition, the spatial network based on RGB pictures can also effectively recognize various kinds of actions with object influence, for example, the action of playing basketball, RGB pictures can improve the recognition accuracy through the recognition of basketball, and then assist in the recognition of the overall action. Among them, the recognition of visual modality is mainly divided into the establishment of gesture recognition model and gesture interaction stage, and the recognition of speech modality is mainly keyword recognition. Finally, a prototype of an intelligent chemistry experiment based on an augmented reality environment is realized by fusing visual, auditory, and haptic information, and its framework diagram is shown in Figure 1.

During gesture recognition, there are errors in skeletal tracking due to differences in human hand size and hand movement in different positions, which affects the accuracy of gesture recognition. Because the magnitude of the hand wave is relatively small, analysis with a single frame is more difficult and less stable, while problems such as self-obscuration can occur. In the feature extraction stage, after obtaining the position of the object and the circumscribed rectangular frame, first perform feature extraction on the object in the circumscribed frame area. The existing frameworks for object recognition can be divided into manual feature-based object recognition framework and deep learning-based object recognition framework in the feature extraction phase (Mohammed and Hassan, 2020). Manual feature-based object recognition framework contains two phases, namely feature extraction and object classification. In the feature extraction phase, after obtaining the location of the object and the external rectangular box, feature extraction is first performed for the objects in the external box region. Shape features are intuitive features that describe objects. Without considering color features and texture features, people can distinguish a variety of objects based on their shape alone. Common object shape features include shape context features, chain code features, area, and horizontal Aspect ratio etc. The quality of the features is crucial to the accuracy of object recognition. Gradient-based HOG features are widely used in object recognition. HOG features divide the region inside the rectangular box into multiple sub-regions and calculate the gradient histogram for each region, and the final feature vector is the combination of the gradient histogram values of multiple sub-regions. In addition to gradient features, object color and object shape are also commonly used to describe objects. Color features mainly describe the color information of a region in an image, where features such as color histogram features and color moments are commonly used. Shape features are intuitive features that describe objects, and without considering color features and texture features, a person can distinguish multiple objects based on object shape alone, and common object shape features include shape context features, chain code features, area, and aspect ratio.

**FIGURE 2:**
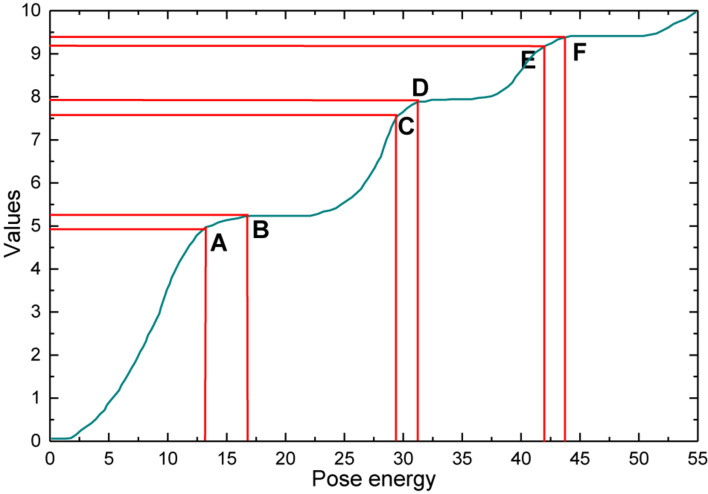
Video segmentation method for artistic pose energy.

Different modal data can provide different information features, while each has advantages for different kinds of recognition. Since depth data is presented as grayscale images and contains the depth information of the video. Therefore, its overall recognition is better than RGB data when recognizing complex actions with less interaction with objects, such as hugging others and shaking hands due to the reason that depth data itself has one more dimensional information than RGB data and possesses the property of robustness to video light and dark, angle transformation, and the absence of complex scene information for that action category. Therefore, in the actual recognition process, this method is likely to reduce the accuracy due to the lack of information between modalities or the redundancy of information. The RGB data, on the other hand, highlights the appearance features of the video data, including features such as color, texture, and shape, as well as information about the human surroundings, as shown in Figure 2.

A video data is divided into three segments by an energy-guided video segmentation method, and each video segment is subsequently randomly sampled to obtain short segments. Where the three video segments are denoted as (*y*_*i*_,*y*_2_,*y*_3_), and the short film segments are denoted as(*x*_*i*_,*x*_2_,*x*_3_). The sampled short segments then fed into the multimodal information complementary network separately to obtain the initial action category scores. The scores of the three short segments are finally fused by weighted fusion to obtain the action category consensus. For the modeling of the short clip segments as shown in Equation (5):


(5) $$f\left(x_{i},x_{2},x_{3}\right)=G(F\left(x_{1};W\right)G(F\left(x_{2};W\right)G(F\left(x_{3};W\right)$$

(6) $$L\,\left(y,g\right)=\sum_{i=1}^{n}y_{i}\,\left(g_{i}+\ln\,\sum_{j=1}^{n}\exp g_{i}\right)$$

But multimodal data not only have information that complements each other but there is also a lot of redundant information between modalities. The feature mapping stitching alone cannot effectively distinguish which features are shared in the feature mapping and which are special features unique to each modality. Therefore, this method is likely to lead to a decrease in accuracy in the actual recognition process due to missing information between modalities or information redundancy (Chen et al., 2020). The eight channels of EMG are used for classification. The subject’s movements are composed of body and gestures. Each movement of the body is before the gesture and the movement is behind. All movements are performed only once in a sample, among which bending movements include bending over and getting up. In addition, in multimodal networks, both low-level features and high semantic features between modalities have complementarity and redundancy, and the pre-fusion strategy cannot effectively share both levels of features at the same time, as shown in Table 1.

**TABLE 1:** Overview of species actions.

**Action number**	**Arm**	**Gesture**	**Action number**	**Arm**	**Gesture**
1	Hold high	Clenched	8	Squat down and raise your hand	Pick up the book
2	Hold high	Right swing	9	Squat down and raise your hand	Pick up the CD
3	Hold high	Scissor hand	10	Squat down and raise your hand	Snap
4	Flat pendulum	Left swing	11	Bend over	Pick up the ball
5	Flat pendulum	Open palm	12	Bend over	Pick up the pen
6	Flat pendulum	Like	13	Bend over	Shoe shine
7	Swing to the chest	Pen cap	14	Sit down	Open the book

Due to the variability of individual subjects, i.e., the subjects themselves have different personal habits, the total duration of each sample varied, and it is not possible to describe the duration of each sample in detail here, but the duration of each action sample did not exceed 15 s. Each action performed three times during the experiment, and for both skeletal and EMG data, the entire segment of each sample was used for classification, and all eight channels of EMG were used for classification. Subjects’ movements were composed of both body and gesture components, with the body movements preceding the gesture movements for each movement. Decrease by 0.55 and 1.48%, respectively. Under the condition of 10-fold cross-validation, the accuracy of MKL with five different initializations for K cluster and the accuracy of MKL with only one initialization for K cluster are both 98.70%. All movements were performed only once in a sample, where bending movements included bending and rising while squatting and sitting movements did not include rising movements.

### Experiments in Human Art Pose Interaction

**FIGURE 3:**
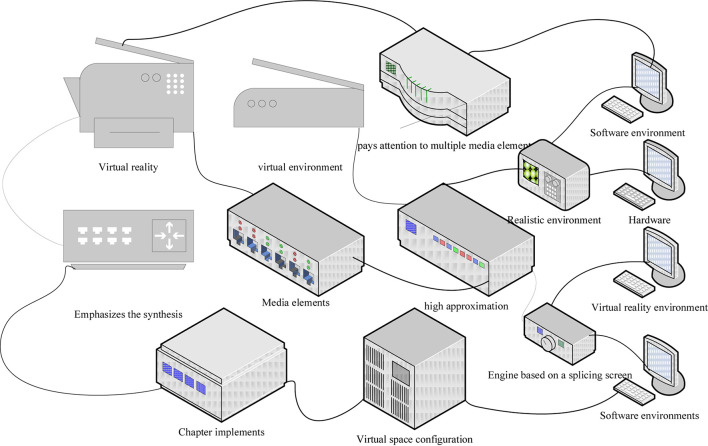
Composition of the experimental environment.

The most important thing about virtual reality is the construction of virtual environment, i.e., virtual world, which not only pays attention to multiple media elements such as text, image, sound, language, animation, etc. but more importantly, emphasizes the synthesis of various media elements to constitute a 3D sensory world with high approximation to the real environment (Qasim et al., 2021). To obtain a better immersion, this chapter implements the construction of a virtual space configuration system in the Unity3D engine based on a splicing screen, Kinect, and a camera. The virtual reality environment contains both hardware and software environments, and the combination of hardware and software environments builds a highly simulated realistic environment that provides a system guarantee for natural interaction. The output of the experimental environment in this paper is a spliced screen solution, which consists of eight screens placed in an arc to ensure immersion when experiencing the virtual environment and portability when navigating the perspective, as shown in Figure 3.

In addition to the use of multimodality and MKL, VLAD features are also helpful. VLAD features encode spatial and temporal information for a given time series using a K-clustering algorithm, which allows features extracted from samples of different lengths to have the same number of dimensions. The dimensionality of the VLAD features extracted from the s EMG signal is 96 and the dimensionality of the VLAD features extracted from the skeletal sequence is 612. This section first analyzes the user’s intention through the expression of modal information. Since there are many different types of virtual experiments, the same type of experiment must be specified when studying the expression of intention. Therefore, we combine the structure and function of the smart device to focus on the intelligent the equipment determines the virtual chemical experiment under a specific interactive situation. In the condition using 10-fold cross-validation, not using intra-cluster principal component analysis and power-law normalization during VLAD extraction leads to a decrease in accuracy of 0.55 and 1.48%, respectively. In the 10-fold cross-validation condition, the accuracy of MKL with five different initializations for K-clusters was 98.70% compared to MKL with only one initialization for K-clusters.

Although more complementary features can be extracted using a K clustering algorithm with multiple initializations, MKL can use weights to measure the contribution of features, which can improve accuracy even with fewer features.

Given the better descriptive power of multi-grain shape descriptors, we combine 17 simple shape features into a robust and accurate shape descriptor that contains both local and global features, reflecting the strong complementarity between multi-grain features, i.e., global features can distinguish widely varying shapes, while detailed differences can be further identified by local features.


(6) $$f_{1}={||p_{i}+g||}_{2}$$

(7) $$f_{11}=\left(\frac{1}{n}\sum_{i=1}^{n}x_{i}^{2},\frac{1}{n}\sum_{i=1}^{n}y_{i}^{2},\right)$$

Wraparound-based feature selection methods can achieve good performance and are easy to implement. We use multiple different wraparound feature selections on the original feature set to filter out several different feature subsets (Ahmad and Khan, 2020). Specifically, to obtain differentiated feature subsets, we subset the feature set using wraparound feature selection methods with different base classifiers. From this perspective, by training on the differentiated feature subsets, differentiated classifier models can be obtained, and then the output of integrating these differentiated models can be used to improve the classification performance in the classifier fusion phase. Then understand the operations performed by the user from this information. In the chemical experiment, the operation requirements and steps are very important. According to the structure, function and gesture types of the smart device designed in this article, combined with the chemical experiment requirements and principles.

Since the wrapped feature selection based on the base classifier does not consider the internal relationship between global and local features, feature selection should be used separately on the global and local feature sets to avoid uninterpretability between global and local features in the filtered feature subset. In addition, for each local feature, feature selection should also be used separately for chain code local features and shape context local features. Specifically, five base classifiers trained by using decision trees, K-nearest neighbors, probabilistic neural networks, fuzzy rules, and random forests can be used to select the five best feature subsets based on the performance of each base classifier. Here the feature selection uses a forward feature search strategy to determine the starting features. After feature selection, the five optimal feature subsets corresponding to the five base classifiers can be obtained.


(8) f=16{C,1C,2…,C}10

(9) F=16{f,1f,2…,f}10

Through technological innovation and design interventions, intelligent machines are made more efficient in recognizing the subtle emotions expressed by users and getting quick understanding. Focus on how the user interacts with the product throughout the design process and apply the principles of good communication to create the desired user emotional experience. Focus on human-centeredness and attention to detail in design to improve the depth and quality of interaction. Combine the context to generate or retrieve the answer, and then synthesize the voice to answer the user through TTS. Design personas and create great experiences through immediate feedback and an emotional engagement approach.

In multimodal interactions, analyzing the relationship between the different modalities corresponding to user actions requires integrating information from all three modalities. Each action behavior of the user is represented as each interaction intention of the user, i.e., the user’s intention is expressed in the interaction behavior (Li et al., 2020). This section first analyzes the user’s intention through the expression of modal information, and since there are many kinds of virtual experiments that exist and must be specific to the same type of experiment when studying intention expression, we combine the structure and functionality of the smart device to identify virtual chemistry experiments for the smart device in a specific interaction scenario.

**FIGURE 4:**
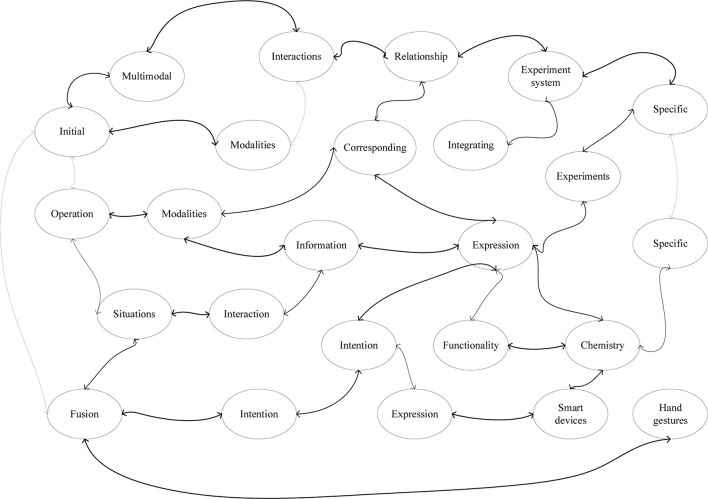
Multimodal information fusion strategy.

The multimodal fusion referred to in this paper is to acquire visual, auditory, and tactile information within a certain period, and then understand the operation performed by the user from this information. The GBT in this experiment did not use any data sampling method, that is, did not use the bootstrap sampling method in the experimental setting, which means that each tree is trained on the same set of samples. In the chemical experiment’s, its operational requirements and steps are very important, according to the structure-function and gesture types of the intelligent devices designed in this paper, combined with the chemical experiment requirements and principles, to accurately simulate the experiment to achieve the effect of the real experiment, and at the same time to meet the user’s intelligent interaction needs, so for the intelligent chemical experiment system, this paper proposes a multimodal information fusion strategy based on directed graphs, we use inputting information from different channels and judging the interaction semantics under different trigger conditions, as shown in Figure 4.

Human-computer voice interaction is landing in increased scenarios, giving rise to a brand-new incremental market. Voice interaction is essentially an interaction mode that uses voice input to understand user needs and provide feedback to them (Saktheeswaran et al., 2020). It first converts the user’s conversational speech into text through ASR, then uses the NLP part to understand the text, generates or retrieves the answer in combination with the context, and then answers the user by synthesizing the voice through TTS. This also shows the superiority of using integrated learning. Although local fusion can effectively improve the recognition rate, the recognition rate after fusion is still lower than the recognition rate using only the RF classifier. At the core of the speech processing module used by Takenuma Intelligence is a mixture of retrieval and generation models. By using the platform provided by the product, enterprise-level and individual-level users generate many conversations and data, annotate general information with specific information protection and confidentiality, and continuously improve the speech and text libraries, allowing users to get increasingly accurate answers in multiple subsequent rounds of conversations.

## Analysis of Results

### Results of Intelligently Enhanced Multimodal Human Art Pose Recognition

Here, we give the setup of each algorithm for each phase. In the feature selection phase, different feature subsets are obtained by wraparound based feature selection methods, which have five different base classifiers, and for the DT classifier, the internal feature selection uses the Gini coefficient and prunes the decision tree to avoid overfitting using the Reduced Error Pruning (REP) method. However, when a feedback mechanism is added, because the mechanism can effectively filter the output results of the target detection network, it adaptively filters out objects and their corresponding tags that are helpful for identifying certain types of actions and corrects the score matrix. The minimum number of records per node is set to 2, and the average split point is selected from the general options. For FR nodes, activation is chosen to use the Min/Max parametrization and the volume border-based shrink function is chosen to reduce the rules to avoid conflicts. For the PNN classifier, theta minus and theta plus are set to default values of 0.2 and 0.4, respectively. For the RF classifier, the information gain ratio is selected as the splitting criterion in the tree option and the integration tree size is set to 100.

**FIGURE 5:**
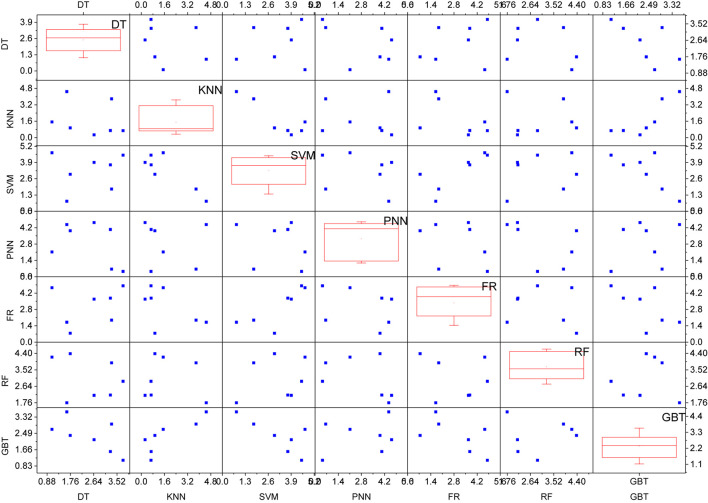
Recognition rate of training classifiers on feature subsets.

For the GBT classifier, the tree depth is set to 10, the number of models is set to 20, and the learning rate is set to 0.1. XGboost is used to handle missing values when dealing with problems for instances belonging to none class. For integrated learning algorithms, data sampling and attribute sampling are very critical. In this experiment, the self-service sampling method is used in the random forest classifier, i.e., the data sampling mode should be set in the experimental setup to be set to random replacement with the same number of instances before and after sampling. The GBT in this experiment does not use any data sampling method, i.e., the self-sampling method is not used in the experimental setup, which means that each tree is trained on the same set of samples. For attribute sampling and selection, each tree in the RF classifier and the GBT classifier uses a different subset of features, with the size of each feature subset being the square root of the total number of attributes. The algorithm has achieved a recognition rate of 91.40% in the MPEG-7 shape data set. First, the key frame of the time series sequence is extracted from the bone data, and then the relative position feature of the bone data is extracted at the key frame to describe the spatiality of the action. For the fusion of all classifiers, the mean value rule is used and each classifier weights 1 as shown in Figure 5.

**FIGURE 6:**
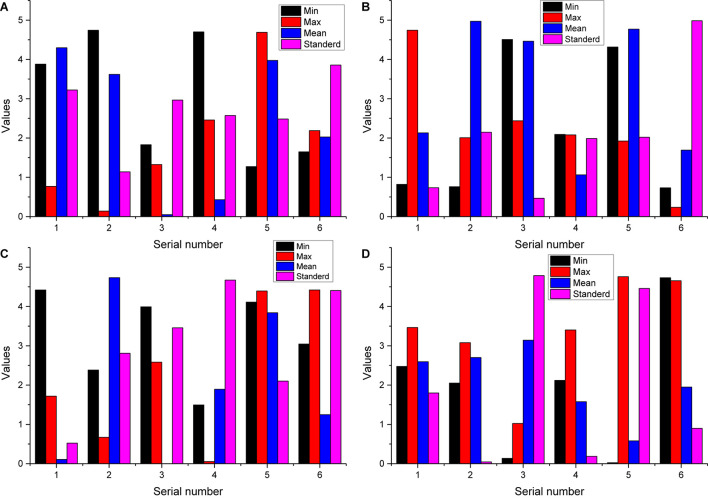
Statistics on measurement questions.

The recognition rates after local fusion by using a subset of differentiated features to train the classifiers were all improved compared to the recognition rates of locally fused individual classifiers. The improvement in recognition rate after local fusion was much more pronounced when DT was used as the base classifier for local fusion (10.2% improvement). For the seven classifiers selected, RF has the highest recognition rate, and it is worth mentioning that RF itself is an integrated learning algorithm, which also indicates the superiority of using integrated learning. Although local fusion can effectively improve the recognition rate, the recognition rate after fusion is still lower than that of using only RF classifiers. There may be several reasons for the above phenomenon: some instances are correctly classified by RF only, and the sum of confidence of other classifiers for that instance is higher than the confidence of RF for that instance during the fusion, thus leading to misclassification of those instances by classifier fusion. Based on the above analysis, if instances that are not correctly classified by the RF classifier are still not correctly classified after classifier fusion, this will lead to an increase in misclassified instances, i.e., a decrease in the overall classification recognition rate, as shown in Figure 6.

**FIGURE 7:**
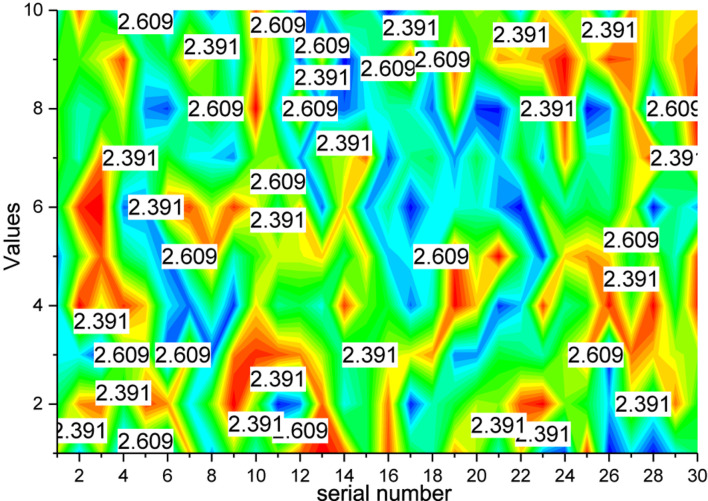
Confusion matrix based on multimodal fusion.

As for the action recognition algorithm module, still taking the cross-topic (CS) NTU data as an example, after training the network with 40,320 training samples, a scoring matrix of size 16,560 × 60 is output for 16,560 test samples, which has 16,560 rows representing the number of video samples, while 60 columns represent the 60 action category scores output, as shown in Figure 7. Finally, the score matrix output by the eye detection network and the score matrix output by the action recognition network proposed by the network fusion strategy are fused and the final fused score matrix is obtained. This fusion score matrix is the final action category discrimination result.

As can be seen in Figure 7, the addition of a feedback mechanism to the network fusion strategy module can effectively improve the accuracy of the action recognition algorithm. However, only converting the output results of the target detection network into the generation matrix without being adjusted by the feedback mechanism will instead backfire on the discrimination of the action recognition algorithm due to the false detection of the target detection network, causing the overall accuracy of the action recognition algorithm to decrease. However, when the feedback mechanism added, because the machine can effectively filter the output results of the target detection network, the objects and their corresponding labels that help recognize a certain type of action are adaptively filtered out and the score matrix is corrected. And when objects that do not help judge a certain type of action are detected, the generation matrix is automatically corrected, which in turn sums up the sum of the two matrices. Traditional action recognition algorithms have low accuracy in recognizing action classes in complex scenes, action classes with sub-action sharing phenomena, and similar action classes with the influence of other objects. The proposed cross-modal cross-fusion strategy, energy-guided video segmentation, and target detection network-assisted algorithms effectively solve the above problems. As can be seen from the figure, the recognition rate of similar action categories in many complex scenes by the conventional network is only around 60–70%.

### Results of the Human Art Pose Interaction Experiment

**FIGURE 8:**
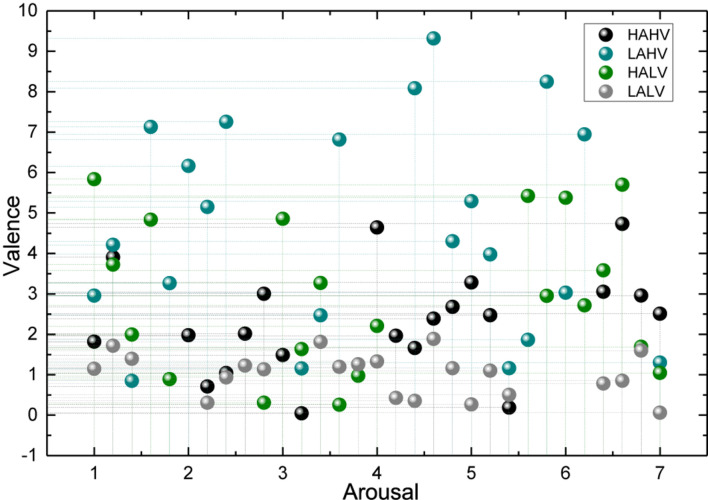
Spatial distribution of subjective emotion ratings of all subjects.

Feature selection and emotion classification were performed for the collected EEG signals and peripheral physiological signals. The subjective emotion ratings of all subjects were used as the reference standard, as shown in Figure 8.

First, in terms of data validity, the performance clips that participated in the experiment carefully selected in this study to elicit the subjects’ emotions as successfully as possible. To prove that the subjects’ emotional experiences were consistent with the emotional ratings of the performance clips, the scores of the 46 subjects’ subjective evaluations were analyzed and compared with the emotional ratings of the performance clips themselves, and the results showed that the subjects’ subjective evaluations were mostly consistent with the emotional ratings of the performance clips, indicating that the emotion-evoking materials were selected more accurately and the subjects could be successfully evoked with the corresponding emotions.

**FIGURE 9:**
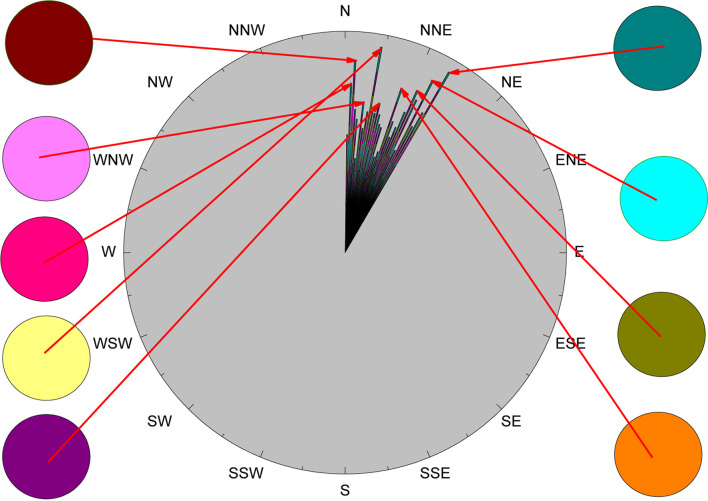
Combinatorial analysis between features.

The LOP area feature counts the number of points falling into the cube, which can roughly describe the area of the object, the LOP orientation feature uses PCA to find the principal direction of all the points in the cube, which can roughly describe the orientation of the object, and the HSV color histogram counts the distribution of the color of the object in the HSV color histogram can count the distribution of the color of the object in HSV space, i.e., describe the color information of the object, and the three features complement each other, as shown in Figure 9.

The number of trees in the random forest will not perform well if the value is too small. As the number of trees increases, the performance of the random forest stabilizes at about 65% after the number is 60 and reaches the highest recognition rate when the number is 120, so this experiment sets the random forest to generate 120 trees for integration. The confusion matrix of the results of this experiment is shown in Figure 8, the recognition results of action 2 and action 5 need to be improved, where the number of correct recognitions of action 1, action 3, action 4, action 6, and action 7 is above 10. Firstly, 66-dimensional features are extracted for the object shape, including global features and local features. Secondly, five different wraparound feature selection methods are used to select five optimal feature subsets from the original feature set, respectively, from the grain computing perspective, and then five homogeneous classifiers are trained on these five optimal feature subsets to merge into a local set, and a total of seven heterogeneous classification algorithms are used to construct seven local sets to merge into a global setting, and the final result output is generated by each classifier voting. The algorithms achieved a recognition rate of 91.40% in the MPEG-7 shape dataset. Keyframe extraction first performed on the temporal sequence by skeletal data, then relative position features are extracted from the skeletal data at keyframes to describe the spatiality of the action, followed by HSV color histogram from RGB data and LOP features from depth data to describe the interacting objects, respectively. Finally, the random forest algorithm used for training and the algorithm was validated on a public dataset.

## Conclusion

This paper proposes a gesture recognition and interaction method based on augmented reality, firstly, the collected gesture map is pre-processed by segmentation, and the gesture recognition model is trained by the deep learning method. Then, through the ARG algorithm, the mapping relationship between hand joint point coordinates and the virtual scene is determined to achieve the effect of virtual-reality fusion between the natural hand and the virtual model. This algorithm solves the coordinate relationship problem between real space and virtual space based on gesture recognition. Its average correct gesture rate reaches 99.04%, which improves the robustness of gesture recognition and proves the effectiveness of this algorithm and the flexibility of gesture interaction. For the recognition and perception methods of the three-modal information, the multimodal fusion method and the overall framework are proposed at the decision level, including the fusion of gesture, speech, and sensor input information. The information of the three modalities is subjected to data set construction and intention analysis, and multimodal fusion is performed by intersecting and merging functions of multimodal information and independent functions to construct a multimodal information fusion interaction strategy to infer the user’s intention and propose a MIDI algorithm. The final validation experiment has a success rate of 92% at normal operation speed, which effectively verifies the feasibility of the method. On the other hand, it demonstrates that multimodality reduces the average operational load by 36% compared to unimodal interaction, i.e., it shows that multimodal interaction reduces the operational load of the user.

## Data Availability Statement

The original contributions presented in the study are included in the article/supplementary material, further inquiries can be directed to the corresponding author/s.

## Ethics Statement

Ethical review and approval was not required for the study on human participants in accordance with the local legislation and institutional requirements. Written informed consent for participation was not required for this study in accordance with the national legislation and the institutional requirements.

## Author Contributions

CM conceived of the presented idea. CM wrote and revised the article. QL and YD provided the suggestions. All authors contributed to the article and approved the submitted version.

## Conflict of Interest

The authors declare that the research was conducted in the absence of any commercial or financial relationships that could be construed as a potential conflict of interest.

## Publisher’s Note

All claims expressed in this article are solely those of the authors and do not necessarily represent those of their affiliated organizations, or those of the publisher, the editors and the reviewers. Any product that may be evaluated in this article, or claim that may be made by its manufacturer, is not guaranteed or endorsed by the publisher.
